# Self‐disclosure about dementia on social media: toward evidence‐informed guidance

**DOI:** 10.1002/alz.091047

**Published:** 2025-01-09

**Authors:** Mahala G English, Mallorie T Tam, Viorica Hrincu, Julie M. Robillard

**Affiliations:** ^1^ University of British Columbia, Vancouver, BC Canada; ^2^ BC Children’s and Women’s Hospital, Vancouver, BC Canada

## Abstract

**Background:**

Social media platforms are increasingly used by people living with dementia and their care partners to seek information and advice, share personal stories, raise awareness, and offer support to others. Engagement with social media is often accompanied by a personal disclosure of a dementia diagnosis or identification as a care partner, but the impact of this disclosure remains unknown. Social media engagement can be beneficial by facilitating peer‐interactions and social support; however experts have raised concerns about the potential for exposure to misinformation and stigma as a result of self‐disclosure. As the popularity of self‐disclosure on social media increases, balancing these risks and benefits is critical to promote healthy and safe social media use for people living with dementia and their care partners. The goal of this project is to deliver an evidence‐based resource to support decision‐making around social media use in dementia.

**Method:**

As a first step toward addressing this goal, the current project aims to identify the motivations and impact of self‐disclosure on social media. Posts related to self‐disclosure were retrieved from Facebook groups and pages over a six‐month period for analysis. Automated and manual sentiment‐ and model‐based interaction analyses were carried out on the data to characterize posts based on their 1) primary motivation for self‐disclosure, 2) polarity, 3) bids for action, and 4) anonymity of the poster.

**Result:**

Preliminary findings reveal information‐ and support‐seeking as the most common motivations for self‐disclosure, highlighting the importance of guidance on identifying misinformation and engaging in healthy peer support.

**Conclusion:**

This work will help empower the dementia community to access support and information safely in the increasingly popular social media spaces to enhance peer supports and access to high‐quality resources.

